# Preferred shallow-water nursery sites provide acoustic crypsis to southern right whale mother–calf pairs

**DOI:** 10.1098/rsos.220241

**Published:** 2022-05-18

**Authors:** Julia M. Zeh, Julia R. G. Dombroski, Susan E. Parks

**Affiliations:** Department of Biology, Syracuse University, Syracuse, NY, USA

**Keywords:** acoustic crypsis, calving habitat, baleen whale behaviour, sound propagation, predator avoidance, eavesdropping

## Abstract

Adaptations to sound production behaviour can reduce the detectability of animal signals by eavesdroppers in a phenomenon known as acoustic crypsis. We propose that acoustic crypsis can include selection of locations that affect how sound transmits through the environment: habitats with poor acoustic propagation can minimize the range of detectability of animal signals. We investigated the potential for the preferred habitats of southern right whales to confer acoustic crypsis. We modelled acoustic propagation and range of detection of calls from southern right whales in the shallow, sandy, near shore waters where mothers and calves aggregate during the calving season. At three nursery sites across three continents in the southern hemisphere, results showed that the depth at which right whales are most commonly sighted has the most limited acoustic detection range for their calls. Thus, these habitats allow mother–calf pairs to remain acoustically cryptic from potential eavesdroppers, both predators and conspecifics, when their calves are the most vulnerable. Our results provide preliminary evidence that, in addition to other behavioural strategies, the use of habitats with poor acoustic propagation can contribute to acoustic crypsis. This adaptation may be a widespread and underappreciated mechanism for avoidance of eavesdroppers.

## 1. Background

Across communication modalities, senders can maximize signal detectability by choosing signalling locations with properties that increase the conspicuousness of their signals. For example, acoustically communicating animals such as frogs, birds and crickets have been documented emitting advertisement signals from locations that favour sound propagation, increasing the range of detection and the chance of detection by a potential mate [1–3]. These locations may be selected over other available sites in the habitat in order to maximize signal transmission to receivers via sound amplification [3], minimal sound degradation [1,2] and/or low noise levels. However, there is often a trade-off between effective transmission of signals to intended receivers and the conspicuousness of those signals to eavesdroppers, which could be conspecifics [4] or predators [5–8]. While there is evidence that signalling location can increase the conspicuousness of acoustic signals, whether senders also choose signalling habitats to minimize signal detectability and increase signal crypsis is less well understood.

Acoustic crypsis refers to behavioural adaptations to acoustic signal production which minimize the potential for detection by eavesdroppers [9]. This phenomenon has been documented across multiple taxa that rely on acoustic communication. Reduced signal amplitude is one behavioural modification used by animals including moths [10], marine mammals [11–14] and birds [15] to avoid detection. Another way to achieve crypsis is to communicate using particular sound frequencies that reduce detectability by eavesdroppers. For example, some odontocetes produce sounds at frequencies outside of the hearing range of their predators or in frequency bands where auditory sensitivity is reduced [8,16]. Additionally, some birds are thought to use high-frequency calls which are difficult for predators to localize [7,17]. A third type of acoustic crypsis is a reduction in call rate or a cessation in acoustic signalling. Reduced call rates and adaptive silence have been observed in insects [6,18], fish [19], frogs [5], birds [20], seals [11] and whales [12–14]. Past studies have focused on these complementary modifications to signal amplitude, frequency and production rate. We propose that in addition to modifications to signal parameters, acoustic crypsis can also be achieved by signalling from a location that limits signal propagation and detection range.

The effects of acoustic propagation can be summarized by the transmission loss, or amplitude reduction, experienced by a sound as it travels. Transmission loss can be used to estimate the amplitude of the sound when it reaches the receiver, which is known as the received level (RL). RL can then be used to estimate the detection range of a sound, or the greatest distance at which a sound is still detectable [21]. For example, high transmission loss would cause a decrease in the amplitude of a sound, which in turn leads to a low RL and a small detection range. Thus, a sound experiencing high transmission loss would only be detectable close to the sound source. Transmission loss and detection range of a signal are determined by the signal characteristics and environmental parameters [21]. In terrestrial habitats, frequency-dependent signal propagation due to environmental filtering has shaped long-range communication signals in a range of taxa [22,23]. In the ocean, these propagation parameters are highly dependent on water depth relative to the wavelength of the signal [21]. Due to interference with both the air–water interface and the sea floor, longer wavelength, low-frequency sounds have limited propagation in shallow waters [21,24]. Thus, changes in water depth can lead to significant changes in the detectability of animal acoustic signals in marine habitats, leading to the potential for acoustic crypsis. Since calving habitats are particularly shallow compared to the other habitats of baleen whale species, we explored the potential for these sites to confer acoustic crypsis by limiting the detection range of acoustic signals in a baleen whale species.

Acoustic signals are used by baleen whales for communication in a variety of contexts, including mate attraction, social coordination and mother–offspring communication [25]. Recent studies have described evidence for acoustic crypsis in baleen whale mother–calf pairs [4,12,13,26]. A combination of quiet calls and low call rates have been recorded from right whale mother–calf pairs (*Eubalaena glacialis & E. australis*) [12–14,27]. These quiet calls may protect vulnerable calves by reducing their detectability by, for instance, adult male conspecifics and killer whales (*Orcinus orca)*. Adult male baleen whales may harass mother–calf pairs, with the potential for physical injury or adverse physiological effects [4,28], and killer whales are the primary predator of baleen whale calves [29,30].

Across continents, southern right whale preferred nursery sites are located in similar shallow, sandy and gradually sloping habitats [31–33]. These characteristics make for poor propagation conditions for low-frequency sounds due to high transmission loss [21,34]. Therefore, we hypothesize that one of the benefits to southern right whale mothers of choosing habitats with these specific oceanographic propagation properties throughout their global distribution is to maximize acoustic crypsis and minimize the risk of eavesdropping during the first few months after birth, when their calves are most vulnerable to predation.

## 2. Methods

**Figure 1.**
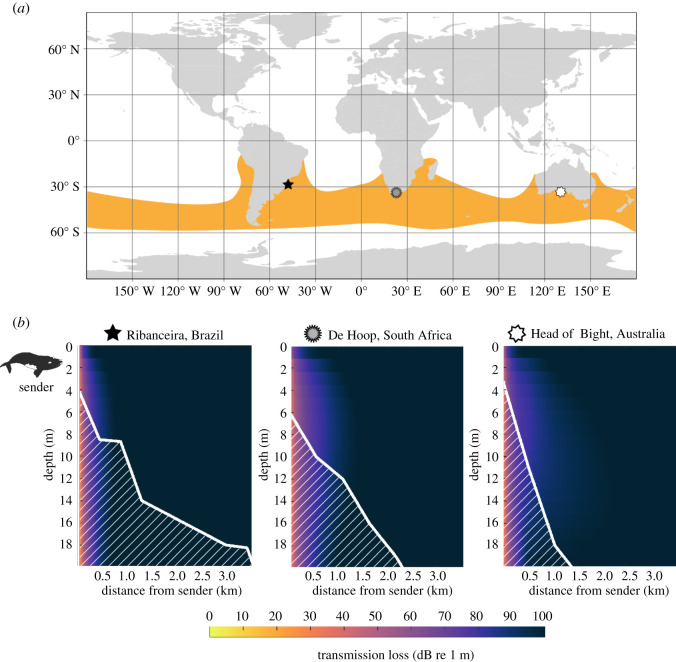
(*a*) The global distribution of southern right whales is shown in orange and the stars show the locations of the three study sites (from left to right: Ribanceira, Brazil, De Hoop, South Africa and Head of Bight, Australia). The world map was created using the R package ‘tmap’ [35] and the southern right whale distribution is based on Jefferson *et al.* [36]. (*b*) Plots of the MMPE outputs showing signal transmission loss over 3 km range for each of the sites marked in (*a*). Model outputs included transmission loss over 5 km, but only the first 3 km are shown in the figure because the maximum transmission loss was reached by then. Stars on the map in (*a*) correspond to stars next to the site names in (*b*). The seafloor is shaded with diagonal lines. The plots show transmission loss of a 102 Hz southern right whale upcall produced at a whale depth of 2 m at the transect point closest to shore (3–6 m water depth). Lower transmission loss indicates a signal would be detectable at greater ranges.

We tested our hypothesis by studying the propagation characteristics of preferred calving habitats across three continents (South America, Africa and Australia), looking at one site for each continent within the global distribution of southern right whales (figure 1). We combined transmission loss modelling with noise level estimates and previously estimated source levels of calls to determine the potential range for detection of calls by eavesdroppers. For each continent, we chose a location within the southern right whale calving habitat where adequate resolution bathymetry data were available: Ribanceira, Brazil [31], De Hoop, South Africa [32] and Head of Bight, Australia [37]. In each of these locations, visual surveys have identified preferred nursery sites as locations with the highest density of mother–calf pairs. These sites are spatially segregated from other whale groups, are close to shore and have water depths between 5 and 10 m [31,32,37–39].

We modelled the propagation of southern right whale calls in shallow near shore waters across all three sites to investigate the potential for acoustic crypsis resulting from habitat propagation characteristics. We modelled the propagation of right whale calls emitted close to shore to reflect the shallow nursery sites where mother–calf pairs are most often sighted. To compare these results to the propagation of calls in deeper waters further from shore, we also modelled the propagation of right whale calls originating at different locations out to 3 km from shore in waters about 18–30 m deep. We were thus able to model propagation at different water depths, testing the hypothesis that preferred nursery sites correspond to areas with poor propagation and consequently reduced detection range and potential acoustic crypsis. To do this, we used the Monterey Miami Parabolic Equation (MMPE) model [40], which models transmission loss with a wave equation solution derived using a split-step Fourier algorithm. We chose this model because it is efficient and accurate in modelling transmission loss in shallow water and range-dependent environments [40,41]. MMPE inputs include signal centre frequency, source depth, range-dependent bathymetry, sound speed profile and seabed properties (table 1; see electronic supplemental materials for MMPE input files and instructions for use). We used a sound source depth of 2 m (a parameter independent of the bathymetry) and a centre frequency of 102 Hz [14].

**Table 1.** MMPE model environmental parameters and references. Range-dependent bathymetry was included in these models, but only start depth and depth at 5 km are listed here. Fine sand (*Φ* = 2.65) and coarse sand (*Φ* = 0.92) definitions are based on Hamilton [42].

	start depth (m)	depth at 5 km (m)	sound speed at 2 m (m/s)	bottom sediment	sediment velocity (m/s)	sediment density (g/cm^3^)	sediment attenuation dB km^−1^ Hz^−1^	references
De Hoop, South Africa	5	32	1507.23	fine sand	1749	1.941	0.5	[42–45]
Head of Bight, Australia	3	37	1507.52	coarse sand	1836	2.034	0.45	[42–44,46]
Ribanceira, Brazil	4	29	1510.37	fine sand	1749	1.941	0.5	[42,44,47,48]

Bathymetry data were obtained from GEBCO, the General Bathymetric Chart of the Oceans [43], for locations in South Africa and Australia and from the Brazil Coastal Modelling System for the Brazil site [47]. We used the ‘marmap’ package in R (R v. 4.0.3, [49]) to visualize bathymetry and create bathymetric transects to use with MMPE [50]. In order to include a range of depths, we created transects that were 5 km in length, oriented approximately perpendicular to shore and located at preferred nursery sites [31,32,37]. Bathymetry data were then entered along the 5 km transect. At each site, we created four 5 km transects with start points at 1 km intervals between shore and a point 3 km from shore. The first transect started within 200 m of 0 m depth (shoreline) in the bathymetry datasets, resulting in an initial depth of 3–6 m. The three other transects started at 1 km, 2 km and 3 km from shore. Sound speed profiles at all sites were obtained from the Global Ocean Sound Speed Profile Library [44]. Sediment type and grain size were obtained for each site and used to define sound velocity in the sediment, sediment density and sound attenuation in the sediment based on Hamilton [42].

We measured ambient noise levels at the Ribanceira, Brazil site with a SoundTrap 300 STD unit (sampling at 96 kHz, 16-bits, −177.2 dB re 1 µPa sensitivity) moored to the sea floor at a depth of 12 m during July/August 2018. Using PAMGuide [51], we calculated 1/3-octave band levels (TOLs dB re 1 µPa) for 24 h of acoustic recordings on a day with calm seas (Beaufort Sea State <2). We used a 1 s-Hann window with 50% overlap and averaged measurements by 3600 s (see electronic supplementary material). We measured ambient noise levels of 106.4 dB re 1 µPa RMS at 100 Hz centre frequency in Brazil, which is similar to the 103 ± 11 dB re 1 µPa RMS noise level in the frequency band of mother–calf calls measured by Nielsen *et al*. [13] at Flinders Bay in the Western Australian breeding ground in calm seas [13]. All of our detection range estimates use the RMS ambient noise levels that we measured in Brazil.

Following previous studies, we estimated detection range using a detection threshold where the RL of a call is equal to the ambient noise level, or the point where the signal-to-noise ratio (SNR) is equal to 0 dB [52,53]. This detection threshold is a conservative estimate and our detection range estimates thus likely reflect a maximum distance at which calls can be detected. To calculate RL, the transmission loss is subtracted from the source level, or the amplitude of the signal at its source (RL = SL – TL). We extracted transmission loss at 2 m depth from the MMPE model output and used source level estimates from the literature (172 dB re 1 µPa; [54]) for our RL calculations. The detection range is then the distance at which the RL reaches the ambient noise level.

The transmission loss model itself is independent of noise level and call source level. Noise level and source level are included here to estimate detection range from the MMPE model output. The high source level used here for southern right whale upcalls (172 dB re 1 µPa; [54]) also makes for a conservative estimate of detection range; given a lower source level, such as that estimated for North Atlantic right whale calls in Parks and Tyack (150 dB re 1 µPa; [55]), the estimated detection ranges would be even lower than those reported here.

## 3. Results

**Figure 2.**
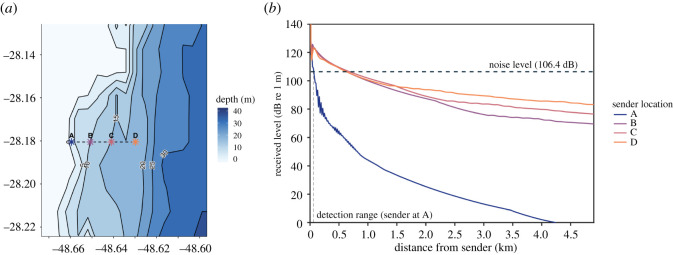
(*a*) Bathymetric map of Ribanceira, Brazil with a dashed line representing the transect used for transmission loss modelling. Southern right whale mother–calf pairs are most commonly observed between points A and B (depth < 10 m), and points C and D are spaced 1 km apart offshore. The colours and letter labels of the points correspond to the colours of the lines and letter labels in (*b*), and these points show the locations used to investigate how propagation changes with bathymetry and distance from shore. (*b*) Plot of RL of a right whale upcall (102 Hz centre frequency) over a 5 km range from the sender location. RL was calculated as the difference between call source level (172 dB re 1 µPa) and transmission loss, which was extracted from MMPE outputs (i.e. Figure 1*b*). The horizontal dashed line shows the ambient noise level at the frequency of the modelled call (TOL 106.4 dB re 1 µPa RMS at 100 Hz). All RLs above the noise level are detectable by a receiver (SNR > 0 dB), whereas those that are below the noise level are not (SNR < 0 dB). The vertical dashed line connects the detection threshold (SNR = 0 dB, RL = NL) and the corresponding detection range (59 m) for a sender at point A on the map in (*a*).

MMPE model results were consistent across all three study sites, showing high levels of transmission loss over a short range for 102 Hz calls produced by a source located within 200 m of shore in very shallow (3–6 m depth) waters (figure 1). Across all three habitats, a 102 Hz right whale call produced close to shore would experience greater than 80 dB transmission loss over a range of 400 m (figure 1). Using a detection threshold where the RL equals the ambient noise level (SNR = 0 dB; NL = 106.4 dB re 1 µPa RMS at 100 Hz), the detection range at 2 m calling depth for a right whale upcall (SL = 172 dB re 1 µPa) is 59 m in Ribanceira, Brazil (figure 2). Using the same parameters, the detection range is 118 m using the transmission loss modelled at De Hoop, South Africa and 74 m using the transmission loss modelled at Head of Bight, Australia. Based on additional transmission loss models for Ribanceira, Brazil, the detection range (distance at which SNR = 0 dB) increases with increasing water depth and sender distance from shore (figure 2; see electronic supplementary materials for additional models at the South Africa and Australia sites). A call originating further from shore (in deeper waters) would experience less transmission loss, resulting in a higher RL. For example, a call produced 3 km further offshore (point D in figure 2) would have a detection range of 618 m, compared to 59 m for a source at point A (figure 2). Southern right whale mother–calf pairs are usually found very close to shore in water depths less than 10 m (i.e. point A in figure 2), a location where transmission loss of their acoustic signals is greatest and the range at which they can be detected is least [32,37–39].

## 4. Discussion

The transmission loss model outputs for southern right whale mother–calf pair vocalizations on the calving grounds suggest that acoustic crypsis to avoid eavesdropping by predators could be facilitated by using habitats with poor acoustic propagation. The calls produced by southern right whales in very shallow water (less than 10 m) located close to shore are unlikely to be detectable by conspecifics or predators that are greater than 500 m away. These model results were consistent across all three study sites across three continents, suggesting that this characteristic may be an important habitat selection factor driving right whale distribution among nursery sites on the calving grounds. Future studies should address whether southern right whale mothers are actively selecting calving habitats with these acoustic propagation characteristics.

Much greater detection ranges have been reported for the upcalls of other right whale species in habitats outside of the calving grounds. For example, maximum detection range estimates were 30 km for North Atlantic right whale calls in the Bay of Fundy [56] and up to 100 km for North Pacific right whale calls on the eastern Bering Sea shelf, due to differences in the propagation characteristics of these habitats [57]. While still considered shallow water habitats (less than 220 m), the locations of these previous studies are an order of magnitude deeper than the nursery sites we investigated. Underwater sound propagation is highly dependent on water depth as it relates to the wavelength of the sound, as well as variations in the sound speed and bottom sediment properties [21], which accounts for these large differences in detection range estimates between our results and those from studies of other right whale species in high-latitude habitats [56,57]. These differences in detection range provide additional support for the hypothesis that right whales may be selecting nursery habitats, at least in part, due to the limited acoustic propagation characteristics when compared to other habitat areas available to them.

Propagation models are generalizations and factors such as tidal range, changes in ambient background noise levels and changes in the call amplitudes by the whales would contribute to variability in the actual detection range in these environments. Near shore habitats, like southern right whale nursery sites, include areas which are exposed at low tide, so the precise location of vocalizing right whale mother–calf pairs and the related water depth varies with the tides. Ambient noise will also vary with environmental conditions, including wind speed and other biological and anthropogenic sources of noise. In turn, right whales are known to compensate for changes in noise level by modifying the amplitude of their calls, a phenomenon known as the Lombard effect [52,58]. However, even accounting for the variability in factors that contribute to detection range, there is a stark contrast in transmission loss between the low-frequency right whale calls in the very shallow water where mother–calf pairs are generally found (depth < 10 m; [32,37–39]) when compared to other available habitat within the calving grounds.

For slowly reproducing baleen whale species like southern right whales, reproduction is costly, and there is strong selection for behaviours which will minimize risk for vulnerable calves [26]. Some of the hypotheses for why baleen whales migrate to low-latitude calving grounds are that these shallow sites have fewer predators and warmer and calmer waters [59]. Our study suggests that another added benefit of these sites is limited acoustic transmission, to allow for mother–calf pairs to stay in close acoustic contact without alerting potential eavesdroppers such as predatory killer whales. In addition to right whales, killer whales are the primary predators for other baleen whale species. This includes humpback whales and grey whales, which both visit seasonal calving sites in shallow, near shore environments [59]. Acoustic crypsis in signal production amplitude, similar to that of right whales, has been observed in migrating humpback whale mother–calf pairs in Australia [4,26]. Additionally, humpback whale mother–calf pairs have been observed moving to shallower waters and spending more time near the surface when there are breeding males nearby [4], behaviours which could allow mother–calf pairs to avoid acoustic detection by conspecifics. Grey whales are known to nurse their calves in shallow lagoons and research has suggested that avoidance of killer whale predators may drive migration to these habitats [60]. Further research on habitat selection, call production and acoustic propagation within the calving habitats of other baleen whale species will aid future comparative studies.

This study provides a first step towards highlighting a fourth behavioural modification that animals can use to minimize acoustic detection. The first modification is a reduction in call amplitude [10,11,13,15] or a switch in call repertoire to lower amplitude call types [12], both of which reduce the detection range. A second modification is to use signal frequencies which are difficult for eavesdroppers to detect and/or localize [7,16,17]. Third, animals may reduce or completely cease acoustic signal production, effectively going silent to avoid detection [5,6,11–14,18–20]. While numerous studies have documented habitat selection to maximize the transmission of signals, for example for defending territories [2] or attracting mates [1–3], we have found little discussion of the opposite approach of habitat selection to minimize the transmission of signals. We suggest that this may be a broadly used fourth behavioural adaptation to avoid detection by predators and suggest that additional research is needed to determine how common a habitat selection approach to acoustic crypsis may be.

## Data Availability

The datasets supporting this article have been uploaded as part of the electronic supplementary material [61].
